# The Application of Machine Learning Models to Predict Stillbirths

**DOI:** 10.3390/medicina61030472

**Published:** 2025-03-07

**Authors:** Oguzhan Gunenc, Sukran Dogru, Fikriye Karanfil Yaman, Huriye Ezveci, Ulfet Sena Metin, Ali Acar

**Affiliations:** 1 Konya City Hospital, Konya 42020, Turkey; oguzhangunenc@hotmail.com; 2 Division of Perinatology, Department of Obstetrics and Gynecology, Medical School of Meram, Necmettin Erbakan University, Konya 42090, Turkey; drkaranfilf@yahoo.com.tr (F.K.Y.); huriyeezveci00@gmail.com (H.E.); 3 Department of Obstetrics and Gynecology, Medical School of Meram, Necmettin Erbakan University, Konya 42090, Turkey; ulfetsenametin@gmail.com (U.S.M.); mdaliacar@gmail.com (A.A.)

**Keywords:** stillbirth, machine learning, prediction, odds ratio

## Abstract

*Background and Objectives*: This study aims to evaluate the predictive value of comprehensive data obtained in obstetric clinics for the detection of stillbirth and the predictive ability set of machine learning models for stillbirth. *Material and Method*: The study retrospectively included all stillbirths followed up at a hospital between January 2015 and March 2024 and randomly selected pregnancies that resulted in a live birth. The electronic record system accessed pregnant women’s maternal, fetal, and obstetric characteristics. Based on the perinatal characteristics of the cases, four distinct machine learning classifiers were developed: logistic regression (LR), Support Vector Machine (SVM), Random Forest (RF), and multilayer perceptron (MLP). *Results*: The study included a total of 951 patients, 499 of whom had live births and 452 of whom had stillbirths. The consanguinity rate, fetal anomalies, history of previous stillbirth, maternal thrombosis, oligohydramnios, and abruption of the placenta were significantly higher in the stillbirth group (*p* = 0.001). Previous stillbirth histories resulted in a higher rate of stillbirth (OR: 7.31, 95%CI: 2.76–19.31, *p* = 0.001). Previous thrombosis histories resulted in a higher rate of stillbirth (OR: 14.13, 95%CI: 5.08–39.31, *p* = 0.001). According to the accuracy estimates of the machine learning models, RF is the most successful model with 96.8% accuracy, 96.3% sensitivity, and 97.2% specificity. *Conclusions*: The RF machine learning approach employed to predict stillbirths had an accuracy rate of 96.8%. We believe that the elevated success rate of stillbirth prediction using maternal, neonatal, and obstetric risk factors will assist healthcare providers in reducing stillbirth rates through prenatal care interventions.

## 1. Introduction

Stillbirth is a serious obstetric result characterized by the death of a fetus at a gestational age of 20 weeks or later or a fetal weight exceeding 500 g [1]. An estimated 1.9 million infants are stillborn each year, and the global burden of stillbirths remains a significant concern for public health [2]. It is crucial to note that the majority of stillbirths (approximately 98%) occur in low- and middle-income countries [3]. Stillbirth has serious medical and psychological effects on parents, as well as financial consequences for healthcare systems and economies [4].

Stillbirth’s etiology varies and can be multifactorial, with maternal, fetal, and placental origins. Maternal risk factors include obesity, nulliparity, advanced age, smoking during pregnancy, a history of stillbirth, preexisting diabetes, and pregnancy-related hypertensive diseases [5]. The most common fetal risk factors for stillbirth include fetal growth restriction (FGR), infection, preterm delivery, and structural or genetic abnormalities [6]. Placental diseases have also been linked to stillbirth [7]. Improved prenatal and intrapartum care, the removal of modifiable risk factors, and the early detection and treatment of congenital defects have significantly reduced the stillbirth rate [8].

Some stillbirths are caused by the failure to identify pregnancies at a high risk of stillbirth and the default in antenatal care, which is expectant management. Studies often restrict the identification of pregnancies at a high risk of stillbirth due to small sample sizes, limited maternal variables, and the exclusion of multiple pregnancies, a recognized high-risk category of potentially preventable stillbirths. Models based on variables not routinely collected in most studies perform poorly in predicting stillbirths and have limited value in guiding decisions during the antenatal care period. Simple linear statistics are insufficient for modeling complex issues like stillbirth. Improvements in computer processing power, memory, and storage, along with the extensive availability of full datasets, have facilitated the implementation of artificial intelligence (AI) and machine learning (ML) in healthcare for enhanced risk prediction. Machine learning can integrate diverse datasets and identify patterns and correlations [9]. ML can enhance early disease prediction, diagnosis, and treatment within maternal–fetal medicine. It has been employed to evaluate fetal well-being and to predict and diagnose conditions. Due to the increasing availability of open-source software and advances in computer science, the opportunity to improve stillbirth prediction through machine learning has emerged. Certain machine learning models more easily exploit the varied and non-linear relationships between risk factors that substantially contribute to the prediction of stillbirth.

The main aim of this study was to evaluate the predictive value of an extensive collection of routinely obtained data for stillbirth detection. The secondary goal of this study is to assess the predictive capability set of machine learning models for stillbirth. This work involves training several machine learning classifiers to predict stillbirth, aiming to achieve optimal accuracy. Each classifier employed in this investigation possesses distinct capabilities. To the best of our knowledge, the machine learning classifiers we employed significantly outnumber the classifiers previously utilized for stillbirth prediction.

## 2. Material and Method

### 2.1. Study Design

This study retrospectively included all stillbirths followed up at the Necmettin Erbakan University Hospital between January 2015 and March 2024, as well as randomly selected pregnancies that resulted in a live birth. Approval for this study was obtained from the university ethics committee with the decision numbered 2023/4504 (application no. 15478). The Helsinki Declaration was followed throughout the study. All patients included in the study had written informed consent. The hospital’s electronic data system provided all pregnancy, fetus, and newborn records.

### 2.2. Participant Selection and Data Collection

Stillbirths are identified as the demise of a fetus that weighs more than 500 g or has a gestational age of 20 weeks or more. According to our definition, living births had a heartbeat and were more than 20 weeks old at the time of birth. This study excluded patients who underwent termination, had multiple pregnancies, experienced intrapartum deaths, or whose delivery records were not available in our clinic. Patients with missing data were excluded from the study.

The consanguinity status of the couples, number of pregnancies, number of births, number of miscarriages, fetal gender, age of the mother at birth, body mass index, smoking status, presence of chronic disease in the mother, history of stillbirth in previous pregnancies, gestational age at birth, in vitro fertilization pregnancies and Rh isoimmunization were evaluated. These characteristics were evaluated considering the demographic and clinical data obtained from pregnancies with stillbirth in previous publications [10,11]. Ultrasound recordings detected fetal anomalies, umbilical cord anomalies, or placental anomalies, as well as growth and amniotic fluid anomalies.

### 2.3. Machine Learning Models

Four distinct machine learning classifiers were developed to predict the likelihood of stillbirth based on the extensive perinatal and maternal data collected. These models included the following: Logistic Regression (LR) [12]: This is a statistical model that is commonly used for binary outcomes, such as the prediction of stillbirth occurrences. Logistic regression assumes that predictors and the log odds of the outcome have a linear relationship. This makes it easier to understand, but it might make it harder to see complex, non-linear interactions in the data. Support Vector Machine (SVM) [13]: This classifier tries to find the best hyperplane for telling the difference between stillbirth and live birth. Support Vector Machines (SVMs) work very well in high-dimensional spaces and with small datasets, but they can have trouble when the decision boundary is very non-linear, which means that the kernel functions need to be fine-tuned very carefully. Random Forest (RF) [12]: This is an ensemble learning technique that constructs multiple decision trees and integrates their outcomes to enhance prediction accuracy and robustness. Each tree is developed using a random subset of the data and features, which mitigates overfitting and enhances generalizability. Random Forest effectively addresses non-linear relationships and feature interactions, rendering it suitable for complex datasets such as those examined in this study. Multilayer Perceptron (MLP): This is a neural network is specifically designed to model complex, non-linear relationships between input variables and outcomes. The choice of hyperparameters, architectural design, and the volume of accessible training data significantly influence the performance of MLPs. Multilayer perceptrons may not work as well as simpler ensemble techniques like Random Forest if they are not tuned well or have access to large datasets.

Data Preprocessing: Before model training, all numerical variables were centered and scaled to standardize the range of features, ensuring that no variable dominated the learning process. Additionally, categorical variables were encoded as binary (0/1) for inclusion in the models. This preprocessing step was essential for optimizing model performance, particularly for machine learning algorithms that are sensitive to the scale of input data.

Train-Test Split: The dataset was split into training and testing sets in an 80–20% ratio. This split allowed the models to be trained on the majority of the data while holding back a portion for independent testing, thus ensuring an unbiased evaluation of model performance.

In this study, for the first step classification task, two classes were considered, namely stillbirth as the positive class and livebirth as the negative class. To evaluate the performance of the classifiers, different performance metrics were calculated including receiver operating characteristics (ROC) accuracy and the Area Under the Curve (AUC). Performance Metrics: In evaluating the performance of each model, several key metrics were used:

- Accuracy: The overall proportion of correct predictions (both true positives and true negatives) made by the model.
- Area Under the Curve (AUC): The area under the receiver operating characteristic (ROC) curve, a widely used measure that evaluates a model’s ability to distinguish between classes (stillbirth vs. live birth). AUC values closer to 1 indicate better discrimination.
- Specificity and Sensitivity: Specificity (the true negative rate) measures how well the model identifies live births, while sensitivity (the true positive rate) assesses its ability to correctly identify stillbirths.
- Positive Predictive Value (PPV) and Negative Predictive Value (NPV): These metrics evaluate the proportion of true positive and true negative predictions, respectively, out of all positive and negative predictions made by the model.

By using multiple performance metrics, the study ensured a comprehensive evaluation of each model’s ability to predict stillbirth, taking into account not just the overall accuracy but also the balance between false positives and false negatives.

### 2.4. Statistical Analysis

SPSS version 20.0 (IBM Corp., Armonk, NY, USA) was employed to conduct all statistical analyses. The Kolmogorov–Smirnov test and histograms were used to evaluate the distribution for normality. The independent sample *t*-test analyzed two independent groups with a normal distribution. In contrast, the Mann–Whitney U test was employed to analyze those with a non-normal distribution. Maternal characteristics were compared between the outcome groups using the chi-square or Fisher’s exact test for categorical variables; the values were expressed as n (%). Univariable and multivariable logistic regression analyses were conducted to identify independent predictors of stillbirth. Variables that showed significant associations in the univariable analyses were included in the multivariable models to control for potential confounding factors. The results were considered statistically significant at a threshold of *p* < 0.05. This study used both univariable and multivariable logistic regression analyses to find the specific maternal and fetal factors that were most important to the risk of stillbirth while taking into account how the other variables interacted with each other.

## 3. Results

### 3.1. Study Participants

This study included a total of 1000 patients. There were 500 stillbirth cases and 500 live birth cases. Among the 500 stillbirth cases, 38 involved multiple pregnancies, 4 involved intrapartum stillbirths, and 6 did not deliver in our clinic, leading to their exclusion from the study. The study excluded one of the 500 randomly selected live births due to missing records. The stillbirth group had a significantly higher BMI, number of previous CS, and number of previous vaginal deliveries than the live birth group (*p* = 0.001). The gestational age at birth was 27.05 ± 5.51 years in the stillbirth group and 37.88 ± 1.86 years in the live birth group (*p* = 0.001). The rate of ambiguous genitalia was high in the stillbirth group (*p* = 0.001) (Table 1).

### 3.2. Stillbirth Prediction

A total of thirty-two sociodemographic and clinical characteristics were evaluated in the study. The consanguinity rate, fetal anomalies, history of previous stillbirth, maternal thrombosis, oligohydramnios, and abruption of the placenta were significantly higher in the stillbirth group (*p* = 0.001). The preeclampsia rate was 8.2% in the stillbirth group and 4.4% in the live birth group (*p* = 0.016). Chronic hypertension was 2.7% in the stillbirth group and 0.8% in the live birth group (*p* = 0.026). Maternal heart disease and hyperthyroidism were significantly higher in the stillbirth group compared to the live birth group (*p* = 0.01, *p* = 0.013, respectively) (Table 2).

**Figure 1:**
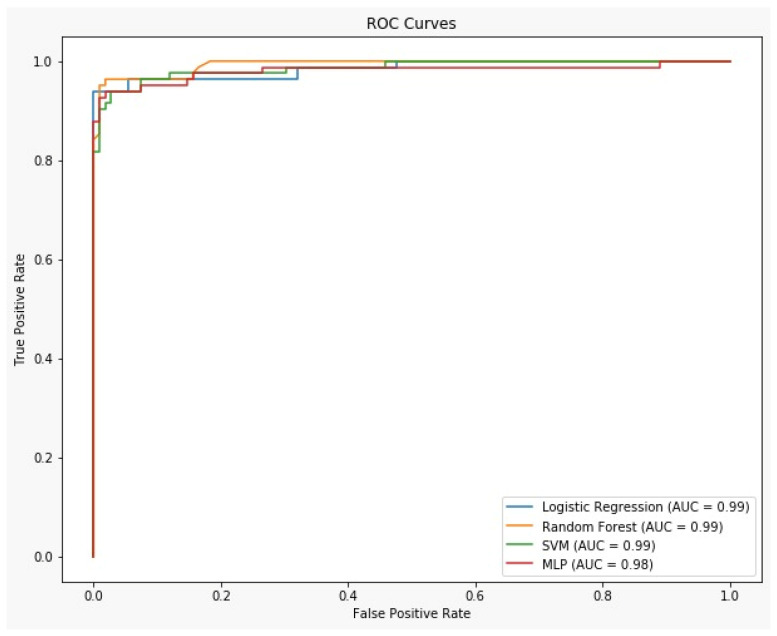
A comparison of the ROC curves of various machine learning methodologies.

Table 3 displays the outcomes of univariable and multivariable regression analyses conducted on the entire cohort of 952 pregnancies. The risk of stillbirth was elevated by the following factors: increased BMI, consanguinity, a history of stillbirth, fetal anomalies, preeclampsia, a history of thrombosis, maternal heart disease, placental abruption, and oligohydramnios. Table 4 shows the accuracy estimates of the machine learning models we used. Accordingly, RF was the most successful model with 96.8% accuracy, 96.3% sensitivity, 97.2% specificity, 96.3% positive predictive value (PPV), and 97.2% negative predictive value (NPV) (Figure 1). RF effectively captures non-linear relationships and intricate interactions among features. RF uses decision trees to divide the feature space into areas that best separate classes, which can handle non-linear data patterns. This differs from LR, which assumes linear data. RF assesses the significance of each feature throughout the training phase, systematically prioritizing the most predictive variables. This capability minimizes the impact of irrelevant or less informative features on the model’s performance. Conversely, MLP models often require significant tuning or regularization to attain comparable robustness. This study utilized a dataset comprising both numerical and categorical variables. RF effectively manages these mixed data types, whereas MLP and SVM may necessitate further preprocessing or encoding to attain comparable compatibility.

A forest plot that shows the odds ratios (95%CI) for the risk of stillbirth based on maternal, fetal, and obstetric characteristics (BMI: body mass index) (Figure 1).

## 4. Discussion

### 4.1. The Study’s Main Outcomes

In this study, we evaluated 452 cases of stillbirth. Increased BMI, consanguinity, fetal anomalies, preeclampsia, a history of thrombosis and stillbirth, maternal heart disease, placental abruption, oligohydramnios, and ambiguous genitalia all increased the risk of stillbirth. We evaluated the potential of a machine learning model for predicting stillbirth. All models had high accuracy, but the RF’s 96.8% accuracy rate was encouraging. RF’s ability to balance sensitivity and specificity while maintaining high accuracy and the AUC makes it ideal for this task. On the other hand, while LR, SVM, and MLP performed adequately, their assumptions, hyperparameter sensitivity, or inability to fully capture the dataset’s complex feature relationships likely hindered them.

### 4.2. Comparative Analysis of Other Studies for Stillbirth Risk Factors

Our research revealed a linear correlation between BMI and stillbirth. This is consistent with the results of other studies that have shown an elevated risk of stillbirth as maternal BMI and weight increase [14,15]. A meta-analysis comprising 38 cohort studies demonstrated a linear relationship between BMI class and stillbirth [16]. The rate of stillbirth is approximately 70% higher in smokers than in non-smokers. According to a systematic review and meta-analysis of studies that involved over 10 million pregnancies, smoking during pregnancy was associated with a 58% increase in the odds ratio of stillbirth at ≥24 weeks [17]. Smoking is believed to increase placental vascular vasoconstriction, which increases the vascular structure’s resistance. A history of venous thromboembolism pre-pregnancy was associated with an increased risk of placenta-mediated complications, including stillbirth, according to a cohort study of 1419 singleton pregnancies [18]. In our study, the prevalence of stillbirth was high in pregnant women with a history of thrombosis (odds ratio [OR]: 14.13). To rule out occult thyroid disease, the RCOG recommends that all women with a late stillbirth have maternal thyroid function tested as part of the stillbirth investigation. However, there is no consensus on the necessity of these tests [19]. A multicenter prospective cohort study of 1025 women did not find a strong association between subclinical thyroid disease and stillbirth. The same study found a correlation between subclinical hyperthyroidism and an increased risk of fetal hydrops-related fetal deaths [20]. Although the stillbirth group in our cohort had significantly higher levels of hyperthyroidism, multivariate analysis did not reveal a statistically significant difference (OR: 1.13, 95%CI:0.73–1.75). In our study, the presence of an umbilical cord knot and cord entanglement in the neck was not significantly higher in the stillbirth group. In contrast, in the meta-analysis of Hayes et al., true umbilical cord knots were associated with an increased risk of stillbirth. Multiple nuchal cords showed a higher rate of stillbirth compared to a single nuchal cord [21]. This result may be attributed to the small patient cohort in our study.

A recent meta-analysis by Deng et al. evaluated 28,322 cases with a history of previous stillbirth. After adjusting for confounding factors, the OR for recurrent stillbirth was 2.68 (95%CI, 2.01–3.56) for women with previous stillbirths compared to women with previous live births [22]. In our series, previous stillbirth histories resulted in a higher rate of stillbirth (OR: 7.31, 95%CI: 2.76–19.31). We may explain this high rate by the fact that our hospital is the tertiary center in the region receiving these patients’ referrals. According to a retrospective cohort study of singleton pregnancies that resulted in stillbirth, the risk of stillbirth was increased by 15 times (OR 15.2, 95%CI 11.0–20.9) when isolated significant congenital anomalies were detected [23]. In total, 10.8% of stillbirths have anomalies, according to the National Vital Statistics Reports, which look into the causes of fetal death [24]. A large study in the United States found that fetal genetic and/or structural abnormalities were responsible for 13.7% of stillbirths [25]. In our series, the OR for the fetal anomaly in stillbirth cases is 8.50 (95%CI: 5.54–13.03). In Rabie et al.’s meta-analysis, stillbirth rates were not evaluated in cases with oligohydramnios because they were found to be very low. In our series, the OR was 2.46 [26]. Casey et al. demonstrated a relationship between stillbirth and oligohydramnios but concluded that the impact of oligohydramnios interventions on the stillbirth rate was unclear [27]. In our series, the fetal gender, whether male or female, did not differ between stillbirth and live-born babies, but the stillbirth group had a high rate of ambiguous genitalia. The associated anomalies explain this scenario. It is still controversial to determine which gender has higher stillbirth rates [28].

A case–control study found that consanguineous marriage was associated with an increased risk of stillbirth. The association was stronger for preterm stillbirths than for term stillbirths [29]. Autosomal recessive disorders and congenital anomalies caused by recessive genes are more prevalent in the offspring of consanguine parents [30]. According to Kapurubandara et al., 1 in every 20 births was to consanguineous couples, and consanguinity was an independent risk factor for stillbirth after controlling for other confounding factors [31]. In our series, the consanguinity rate for stillbirth was high (OR: 6.14, 95%CI: 2.80–13.43). This may be attributed to the high rate of consanguineous marriages in Middle Eastern countries like ours.

Globally, hypertensive disorders of pregnancy (HDPs) are responsible for 15% of perinatal deaths. Pregnancies troubled by HDP account for approximately 16% of the 2.6 million stillbirths that occur annually [32,33]. All of these studies have demonstrated that women with HDP have a higher risk of stillbirth than normotensive women. This could be due to ischemia in the placenta, associated with HDP [34].

### 4.3. Stillbirth Prediction with Machine Learning in the Literature

Given the impact of stillbirth, it is crucial to be able to accurately predict this condition. Previous methods for stillbirth prediction, such as statistical modeling, may be inadequate in their ability to assess the multifactorial determinants of stillbirth and the complex interactions between potential risk factors. Models based on machine learning do not necessitate the prior selection of predictors. They can automatically and thoroughly investigate the complex connections and interactions between potential risk factors and outcomes. The selection of features significantly influences the accuracy and efficiency of predictive machine learning models, necessitating the careful choice of input features to balance accuracy with limitations in data [35]. This investigation aims to optimize the accuracy of stillbirth prediction by training and employing a variety of machine learning classifiers. In our series, the use of measurements collected throughout pregnancy resulted in an accuracy of 96.8% for RF. The most important variables were a previous history of thrombosis and stillbirth, placental abruption, the presence of an anomaly, and consanguinity. These findings provide an important first step in creating a common risk calculator to accurately classify those at increased risk of stillbirth. Regarding the prevention of stillbirths, this strategy is highly effective in identifying those who are at risk. Several machine learning methods are employed to evaluate the significance and relevance of each feature. In the past, logistic regression (an 88% accuracy rate) and other approaches that depend on linear connections between variables have been employed to predict and thereby avoid stillbirth [36]. Other researchers have attempted to utilize machine learning for predicting stillbirth. However, these models have certain limitations in terms of accuracy and the availability of variables. Khatibi et al. used complex feature selection strategies, such as K-means cluster analysis, to find important features and add them to machine learning classifiers. These authors achieved a model accuracy of up to 90.6%, but they did so using measurements such as gestational age at birth, which were not significant in predicting prenatal stillbirths in these data [37]. Malacova et al. attained a peak accuracy of 84% by utilizing measures obtained throughout the ongoing pregnancy as well as the mother’s medical and obstetric information [38].

Higher-order predictors of live birth and prenatal stillbirth should be classified as controllable and unmanageable variables. For controllable variables, suitable policies may be implemented to manage and reduce them, preventing or at least reducing the occurrence of stillbirth. Future research should concentrate on assessing the effectiveness of various strategies used for the monitoring and management of high-ranking predictors of stillbirth.

### 4.4. Limitations

This study’s single-center design is one of its limitations. However, we created comprehensive profiles of all eligible stillbirth cases in this study by conducting detailed chart reviews, which would be very difficult in a population-based evaluation. Other limitations include a lack of knowledge about pregnant women’s socioeconomic status, a lack of validation studies, prenatal care, and educational level, as well as the exclusion of pregnant women of different races. These findings need to be generalized across different populations and healthcare settings.

## 5. Conclusions

Using an extensive set of stillbirths and live births, we used machine learning techniques to develop a pilot model with a 99% AUC to predict stillbirths. The high success rate of stillbirth prediction using maternal, neonatal, and obstetric risk factors is encouraging in terms of reducing stillbirth rates through prenatal care precautions. We believe prospective studies should confirm this dataset and our accuracy rates.

## Figures and Tables

**Table 1:** The clinical and sociodemographic characteristics of the study groups.

Parameters		Stillbirth Group (n:452)	Live Birth Group (n:499)	*p* Value
Maternal age		30.22 ± 6.33	30.24 ± 5.84	0.961 *
BMI (body mass index)		28.84 ± 3.28	27.70 ± 4.62	0.001 *
Gravida		3 (1–10)	3 (1–11)	0.802 **
Parity		1 (0–7)	1 (0–6)	0.647 **
Previous miscarriage		0 (0–7)	0 (0–7)	0.633 **
Previous cesarean section count		0.78 ± 0.98	0.54 ± 0.90	0.001 *
Previous vaginal delivery count		0.56 ± 0.99	0.79 ± 1.13	0.001 *
Induction methods used	Oxytocin	86 (19%)	34 (6.8%)	0.001 ***
	Misoprostol	164 (36.3%)	34 (6.8%)	0.001 ***
	Oxytocin + misoprostol	24 (5.3%)	34 (6.8%)	0.333 ***
Birth week		27.05 ± 5.51	37.88 ± 1.86	0.001 *
Delivery type	VD	303 (67%)	100 (20%)	0.001 ***
	CS	149 (33%)	399 (80%)	
Gender	Girl	187 (41.4%)	237 (47.5%)	0.223
	Boy	242 (53.5%)	261 (52.3%)	
	Ambiguous genitalia	23 (5.1%)	1 (0.2%)	0.001 ***

Bold means statistically significant. * Independent *t*-test, ** Mann–Whitney U, *** chi square, VD: vaginal delivery, CS: cesarean section.

**Table 2:** Prediction of stillbirth by maternal, fetal and obstetric features.

Parameters			Stillbirth Group (n:452)	Live Birth Group (n:499)	*p* Value
Consanguinity		Present	44 (9.7%)	10 (2%)	0.001 ***
Fetal anomalies		Present	150 (33.2%)	34 (6.8%)	0.001 ***
Primiparity		Present	153 (33.8%)	146 (29.3%)	0.128
IVF		Present	10 (2.2%)	14 (2.8%)	0.560 ***
RH/rh		Present	31 (6.9%)	49 (9.8%)	0.100 ***
Stillbirth history		Present	35/7.7%)	7 (1.4%)	0.001 ***
Cigarette		Present	4 (0.9%)	0 (0%)	0.035 ***
PPROM		Present	62 (13.7%)	52 (10.4%)	0.118 ***
Preeclampsia		Present	37 (8.2%)	22 (4.4%)	0.016 ***
Chronic hypertension		Present	12 (2.7%)	4 (0.8%)	0.026 ***
Pre-existing diabetes mellitus		Present	16 (3.5%)	27 (5.4%)	0.166 ***
GDM		Present	11 (2.4%)	20 (4%)	0.172 ***
Cholestasis		Present	1 (0.2%)	1 (0.2%)	0.944 ***
History of thrombosis		Present	49 (10.8%)	6 (1.2%)	0.001 ***
Maternal heart disease		Present	17 (3.8%)	6 (1.2%)	0.010 ***
APS		Present	1 (0.2%)	3 (0.6%)	0.366 ***
Placenta previa		Present	18 (4%)	12 (2.4%)	0.165 ***
Ablatio placenta		Present	16 (3.5%)	2 (0.4%)	0.001 ***
Placenta accreta spectrum		Present	5 (1.1%)	5 (1.0%)	0.875 ***
Polyhydramnios		Present	17 (3.8%)	17 (3.4%)	0.769 ***
Oligohydramnios		Present	103 (22.8%)	63 (12.6%)	0.001 ***
Fetal growth restriction		Present	62 (13.7%)	51 (10.2%)	0.096 ***
Cord prolapse		Present	1 (0.2%)	1 (0.2%)	0.944 ***
Cord knot		Present	1 (0.2%)	0 (0%)	0.293 ***
Nuchal cord		Present	11 (2.4%)	8 (1.6%)	0.361 ***
Thyroid disease	Hypothyroidism	Present	59 (13.3%)	71 (14.3%)	0.678 ***
	Hyperthyroidism	Present	10 (2.5%)	2 (0.5%)	0.013 ***

IVF: in vitro fertilization, PPROM: preterm premature rupture of membrane, GDM: gestational diabetes mellitus, APS: antiphospholipid syndrome, *** chi-square. Values in bold represent statistical significance (*p*-value < 0.05).

**Table 3:** A univariable and multivariable logistic regression analysis was performed to predict stillbirth using maternal, fetal, and obstetric characteristics.

	Univariable		Multivariable	
Variable	OR (95%CI)	*p*	OR (95%CI)	*p*
BMI	1.07 (1.03–1.11)	0.001 *	1.07 (1.03–1.12)	0.001 *
Consanguinity	5.27 (2.62–10.61)	0.001 ***	6.14 (2.80–13.43)	0.001 ***
History of stillbirth	5.89 (2.59–13.42)	0.001 ***	7.31 (2.76–19.31)	0.001 ***
Fetal anomalies	6.79 (4.55–10.12)	0.001 ***	8.50 (5.54–13.03)	0.001 ***
EMR	1.36 (0.92–2.02)	0.119		
Preeclampsia	1.93 (1.12–3.33)	0.018 ***	2.12 (1.13–3.95)	0.024 ***
Chronic hypertension	3.37 (1.08–10.54)	0.036 ***	3.25 (0.82–12.85)	0.092 ***
Diabetes mellitus	1.55 (0.829–2.93)	0.169		
GDM	1.67 (0.79–3.53)	0.176		
Cholestasis	1.10 (0.06–17.70)	0.944		
History of thrombosis	10.84 (4.29–27.37)	0.001 ***	14.13 (5.08–39.31)	0.001 ***
Maternal heart disease	3.53 (1.31–9.55)	0.013 ***	3.79 (1.25–11.42)	0.018 ***
Hyperthyroidism	5.56 (1.21–25.54)	**0.027**	1.13 (0.73–1,75)	0.576
APS	2.72 (0.28–26.31)	0.386		
Placental abruption	9.11 (2.08–39.88)	0.003 ***	12.76 (2.28–71.22)	0.004 ***
Placenta percreta	1.15 (0.29–4.46)	0.837		
Polyhydramnios	1.02 (0.47–2.24)	0.945		
Oligohydramnios	2.56 (1.77–3.70)	0.001 ***	2.46 (1.65–3.65)	0.001 ***
Fetal growth restriction	1.36 (0.89–2.09)	0.153		
Nuchal cord	1.53 (0.61–3.84)	0.364		

BMI: body mass index, EMR: early membrane rupture, GDM: gestational diabetes mellitus, APS: antiphospholipid syndrome. * Independent *t*-test, *** chi-square. Values in bold represent statistical significance (*p*-value < 0.05).

**Table 4:** Stillbirth predictive analysis by machine learning models.

	Logistic Regression	Random Forest	Support Vector Machine	Multi-Layer Perceptron
Accuracy	94.24%	96.86%	95.29%	94.24%
Sensitivity	93.90%	96.34%	91.46%	92.68%
Specificity	94.50%	97.25%	98.17%	95.41%
PPV	92.77%	96.34%	97.40%	93.83%
NPV	95.37%	97.25%	93.86%	94.55%
AUC (%95 CI)	0.98 (0.96–1.00)	0.99 (0.97–1.00)	0.98 (0.96–1.00)	0.97 (0.95–1.00)

PPV: positive predictive value, NPV: negative predictive value, AUC: Area Under the Curve.

## Data Availability

Data are available on request due to privacy/ethical restrictions.
